# Evaluation of the Effectiveness of a Positive Youth Development Program for Secondary Students in Macau

**DOI:** 10.1100/2012/621841

**Published:** 2012-07-31

**Authors:** Andrew L. Luk, K. M. Leong, Annah M. L. Au

**Affiliations:** Kiang Wu Nursing College of Macau, Macau

## Abstract

A well-tested comprehensive Chinese positive youth development program (Project P.A.T.H.S.) developed in Hong Kong has been modified and adapted for use in Macau. This program aims to help adolescent school children develop positively and to be better prepared for their future. The present study investigated the effectiveness of the Tier 1 Program of “P.A.T.H.S.” for Secondary 2 students of two pilot schools. Since there were “repeating” and “transferring” students joining the program, the effectiveness of the program on these particular groups of participants was also examined. The subjective outcome evaluations including participants' perceptions of the program, program instructors, benefits from the program, and overall satisfaction were positive. Although the longitudinal data from the objective outcome evaluation did not show any notable improvement, the overall effect of the program was found to be positive to the new comers in the junior secondary years. The existing evaluation findings suggest that the Secondary 2 program is especially effective to those newly joining the program. In view of the paucity of youth studies in Macau, the present study can contribute to evidence-based youth work and provide baseline data for the program to be evaluated in the Secondary 3 periods in the future.

## 1. Introduction

Macau is a small city located near Hong Kong in South East Asia, famous for tourism and an increasing gaming industry. In 2010, the estimated population of Macau was 542,400, and it has a comparatively young population with those aged between 10 and 24 years contributing 22 percents to the total population [1]. The Macau Government opened the gaming licensure in 2002, and this leads to a rapid development of this industry, generating a big increase of revenue and contributing to the economic growth of Macau. However, this may also bring with it potentially negative influences on adolescents. Due to employment opportunities and perks in the gaming industry, many adults work within the casinos, and this requires them to work long and irregular hours. One potential implication of this development was highlighted in a recent government report which clearly identified the problem on lack of communication of parents with their children and the adverse effect on their adolescent development [2]. The Youth Indicators published by Education and Youth Affairs Bureau in 2009 revealed that many teenagers lacked social norms and their participation in social functions/affairs, and the sense of belongingness to Macau had deteriorated in comparison to an earlier study conducted in 2006 [3]. This research also reflects a dramatic increase in youths stress levels, originating from pressure at school as well as family conflicts. A recent study has revealed that over half of 744 respondents (54%) agreed that gambling was a common phenomenon in young people in Macau. It is suggested that, by building up positive social norms and a sense of morality in adolescents, a more harmonious society in Macau may be resulted [4].

A well-structured local youth program can potentially help adolescents develop positive growth and ensure that they are better prepared for future challenges in life. At present, youth studies and theoretically sound and comprehensive programs for adolescent positive growth and development in Macau are lacking. [5, 6]. Hong Kong and Macau share a similar Chinese culture; therefore, the well-tested comprehensive Chinese positive youth development program “P.A.T.H.S.” developed in Hong Kong [7, 8] has been modified and adapted for use in Macau. The acronym “P.A.T.H.S.” denotes Positive Adolescent Training through Holistic Social Programs. There are two tiers of programs (Tier 1 and Tier 2) in this project. Tier 1 program is a universal positive youth development program in which students in Secondary 1 to 3 participate normally 20 hours of duration or at least with 10 hours of training of the core program during each academic year. With the support from the Education and Youth Affairs Bureau, a local research team was formed by the author and his colleagues who modified the program with some changes of the content relating to local terminology, government structure, and indigenous customs [9]. The team also monitored the implementation of the program and evaluated its effectiveness after the completion of the program for three consecutive academic years. Two secondary schools were invited to participate as pilot schools to run the program starting from their Secondary 1 students. Training for teachers and school social workers was also organized both in Hong Kong and Macau. The initial findings of this Secondary 1 program evaluation was completed during the academic year 2009-2010 and reported positively [9].

Macau has a history of a 15-year free nontertiary education system with direct promotion from primary to secondary school without any public examination. Individual admission examination is required after secondary school education for entry into local universities. Consequently, there is a rather relaxed and less competitive learning atmosphere in the education system. It is not uncommon to see students repeating their studying in some classes in the primary and secondary schools if they find their academic performances unsatisfactory. It is roughly estimated that about 20% of students may repeat their studying in the junior secondary years in many schools [10]. In this paper, the effectiveness of the Macau version of the Tier 1 Program of “P.A.T.H.S.” for Secondary 2 students in 2 pilot schools during the academic year 2010-2011 is evaluated. Both subjective and objective outcomes were measured. Since there were imported students either repeating the Secondary 2 class or transferred from other school joining the Secondary 2 classes of the pilot schools, the effectiveness of the program on these new participants was also examined.

## 2. Method

### 2.1. Participants and Procedures

The study participants included all Secondary 1 students in the two chosen schools, totally 232 starting from year 2009, these were followed up for three years up to Secondary 3. When this group of students was promoted to Secondary 2 year, 43 of them dropped out of their classes and 79 new students either repeating in Secondary 2 or transferred from other schools had joined the program. Data was collected at two time-points during each year. Firstly, before the program started, pretest self-reported questionnaire was completed within 1-2 weeks after the start of the school year. The second data collection time-point occurred after the students finished the program at the end of that academic year. The pretest data Wave 1 (W1) and posttest data Wave 2 (W2) of Secondary 1 had been collected and analyzed [9]. This data was used as baseline for the longitudinal assessment for the Secondary 2 year. The pretest data Wave 3 (W3) and posttest data Wave 4 (W4) of Secondary 2 were collected in this study to assess the effectiveness of the Secondary 2 program.

At pre- and posttests, the participants were invited to complete a valid and reliable questionnaire including measures of positive youth development, life satisfaction, school adjustment, adolescent problem behaviors, and demographic information. An identical questionnaire was used in the pre- and posttests, the paired-samples *t*-test were performed to examine the pre- and posttest differences between the scales, providing an objective outcome measures for evaluation. After completion of the program each year, an evaluation questionnaire was also completed by the participants to assess their satisfaction with the course and perceived benefits of the program, providing subjective outcome measures for evaluation.

### 2.2. Instruments

The two sets of questionnaires which were used in Year 1 were used again when the students were promoted to Year 2. The components in these questionnaires are described below.

### 2.3. The Chinese Positive Youth Development Scale (CPYDS)

The Chinese Positive Youth Development Scale (CPYDS) is a self-administrated questionnaire developed by Shek et al. [11]. It consists of 15 subscales (90 items) which address the 15 constructs of the program. These subscales include bonding (BO, 6 items), social competence (SO, 7 items), emotional competence (EC, 6 items), cognitive competence (CC, 6 items), behavioral competence (BC, 6 items), moral competence (MC, 6 items), self efficacy (SE, 7 items), prosocial norms (PN, 5 items), resilience (SE, 6 items), self-determination (SD, 5 items), spirituality (SP, 7 items), identity or clear and positive identity (ID or CPI, 7 items), beliefs in the future (BF, 7 items), prosocial involvement (PI, 5 items), and recognition for positive behavior (RP or PB, 4 items). The instrument had a good reliability (alpha = 0.91) and ranged from 0.63 to 0.86 [12]. The Cronbach's alpha of the present study is 0.93 and ranged from 0.62 to 0.87. A higher score indicates a higher level of positive youth development.

### 2.4. Life Satisfaction Scale (LIFE)

Life satisfaction is another important indicator of positive youth development [13]. The five-item LIFE was developed by Diener et al. [14] to assess one's own global judgment of one's quality of life. The Chinese version was translated by Shek [15] with acceptable psychometric properties. The Cronbach's alpha of the present study is 0.80. A higher LIFE scale score indicates a higher level of life satisfaction.

### 2.5. Behavioral Intention Scale (BI)

The five-item scale was used to assess the adolescents' behavioral intention to engage in problem behavior, including drinking, smoking, taking drugs, having sex with others, and participation in gambling. The scale has a good reliability (alpha = 0.84) [16]. The Cronbach's alpha of the present study is 0.71. A higher BI scale score indicates a higher behavioral intention.

### 2.6. School Adjustment Measures (SA)

The school adjustment measures include three items. Two items assess the participant's perception of his/her academic performance. The third item assesses the participant's perception of his/her conduct. Previous studies showed that these measures were temporally stable and valid [17, 18]. The Cronbach's alpha of the present study is 0.84. In line with other measures, a higher scale score indicates a higher level of school adjustment in this study. 

### 2.7. Subjective Outcomes Scale (Form A)

The Subjective Outcome Evaluation Form (Form A) was designed by Shek and Siu [19]. The Form consists of totally 39 items and four open questions which are divided into five parts. The first part asks for the participants' views on the program (10 items). The second part looks at the views of the participants towards those involved in delivering the program, including teachers and/or social workers (10 items). The third portion examines the participants' views about their perceived effectiveness of the program (16 items). Three items ask about tendency of participants to join a similar program in the future, their overall satisfaction with the program, and whether they would recommend the program to others. The final part consists of four open questions on things that participants have learned and appreciated most, as well as opinions about the instructors and areas for improvement, respectively. The form had a good reliability on 39 items (alpha = 0.99, mean interitem correlation = 0.80) [20]. The present study has a Cronbach's alpha of 0.98 with mean interitem correlation 0.55. 

## 3. Results

### 3.1. Demographic Information

In total, there were 268 and 244 students who participated in the Wave 3 pretest and Wave 4 posttest, respectively. Out of 268 Secondary 2 students, almost one-third of them, 79 (29.48%) were students who were new to the program. Once invalid questionnaires were discarded, mainly due to missing data, 236 questionnaires were appropriately completed for analysis. There were 173 old participants and 63 new participants (Table 1). Results showed that, with the exception of age, there were no statistically significant differences in sociodemographic background between the old and new students using the chi-square test. The mean age of the new students (*μ* ± SD = 15.08 ± 0.87) was higher than that of the old group (*μ* ± SD = 13.87 ± 1.11). Details can be seen in Table 2.

### 3.2. Subjective Outcome Evaluation

Table 3 highlights the participants' perception of the program and program instructors. Firstly, more than two-thirds of the participants viewed the program positively. For example, 77.4% of the students indicated that the objectives of the program were very clear; 77.8% felt that they had participated actively during the program; 69.0% had a very positive evaluation of the program. Secondly, more than four-fifths of the students provided a positive evaluation of their program instructors. The vast majority of participants (84.7%) felt they were well cared for, and 83.1% of them provided overall positive evaluation of the instructors. The participant's perception of the benefits of the Tier 1 program revealed the following findings. Firstly, more than two-thirds of the participants perceived the program was effective for their personal development and enhanced their psychosocial competences (Table 4). In addition, they thought it also enriched their overall development (73.9%). Secondly, a majority of respondents (63.2%) indicated that they would recommend the program to their friends, and more than a half of them (51.3%) would join similar programs in the future (Table 5). Finally, more than two-thirds of the participants indicated that they were satisfied with the program (Table 5). The qualitative analysis of the four open-ended questions will not be reported in this paper.

### 3.3. Objective Outcome Evaluation

The overall evaluation of all Secondary 2 students, using the paired *t*-test to compare the W3 and W4 data of the program, revealed that there was a nonsignificant decrease in the score of CYPDS, SA or increase of LIFE. Besides, there was a significant slight increase in the score of BI (Table 6). To assess the continual effect of the program from Secondary 1 to Secondary 2, the W1 data of the Secondary 1 program was used as a baseline to measure the longitudinal effect after the participants had completed the Secondary 2 program. Only 189 participants completed Secondary 1 and Secondary 2 programs, using the repeated ANOVA for W1, W3, and W4 data revealed similar nonsignificant results. However, comparison between the W3 data of old participants with new participants revealed that the old students demonstrated better development, life satisfaction, and school adjustment than the new students. Significant differences were reported within the subscales of CYPDS, LIFE, and SA scores (Table 7). As there was a significant difference of age between the groups, analysis of covariance was conducted using age as a covariate. A statistical significant difference of ID subscale and LIFE score was also found. As shown in Table 8, when using the pair *t*-test to compare the W3 and W4 data of the new participants, results showed that there was significant improvement in EC, CC and ID subscales, LIFE, and SA scores. However, there was a significant decrease in BO subscale and an increase of score of the BI scale.

## 4. Discussion

In this study, the participants' perception of the program and their program instructors were evaluated positively. With regard to their perception to the effectiveness of the program, and the overall satisfaction, results provided a positive feedback. Overall, subjective outcome evaluation generally supported positive perceptions to the program, program instructors, benefits from the program, and overall satisfaction with the whole course, these findings are consistent with those reported in Hong Kong [20, 21]. However, when asked about whether they would join similar program in the future, just over half of the participants replied positively. There may be several possible reasons for this dissociation between individuals' perception towards the program and their behavioral intention to participate. Firstly, as suggested, students might think that similar programs may contain similar elements and not be motivated to join such programs again [20]. In fact, the three-year program is designed with the same 15 constructs but with more in-depth exploration of the constructs for senior classes. Secondly, presently students are more prone to be interactive in class. As revealed in the previous study by Luk et al. [9] on the Secondary 1 students, many of them felt that the program was boring. Therefore, if they have a choice, they may not be willing to join even if it may be beneficial for them. Thirdly, when students move to Secondary 3 classes, there are likely to be higher academic demands placed on them. Moral class or personal growth courses like P.A.T.H.S. may not be seen by the students as a high priority.

With regard to the objective outcome evaluation, our findings showed no significant improvement in the Secondary 2 program at posttest, which is inconsistent with findings in Hong Kong [22]. There may be several possible reasons. Firstly, the number of participants in this study is relatively small in comparison to the Hong Kong study which had 20 experimental schools with 2,784 students. This potentially increases the chances of sample bias in the study. Secondly, instructors in the Macau program included social workers and teachers, and these professions are relatively new to this type of interactive youth program, which may affect their effectiveness in conducting the program. Thirdly, the instructors also have to adapt the modified program to meet their student needs, and they may take time to practice and become familiarized with the program. Finally, a certain number of students dropped out and exchanged with a group of new students joined in may affect the group cohesion which in turn may affect their involvement in the program.

However, when the W3 pretest data of the old participants were compared with those of the new inexperienced participants, the findings generally showed that the old students performed better than the new ones. This was evidenced in terms of the global positive youth development and school adjustment indicators. Even with control for age, results still revealed that there was an improvement in positive identity subscale and life satisfaction scale. This comparison may demonstrate the effectiveness of the Secondary 1 program. Furthermore, when comparing the W3 and W4 data of this new group of students, significant improvement in different youth subscales, life satisfaction, and school adjustment measures was noted. This evidence supports the effectiveness of the Secondary 2 program in this group of students. One possible explanation may relate to the nonacademic nature of the program, so it may be more receptive to those whose academic performance is of average or below academic standard. As this group of students is older than the students who were promoted from their original schools, most of them are probably of average or lower standard. Another reason may relate to the fact that this is a new program and may attract their interest more than the older students. Conversely, the decrease in bonding score could be attributed to taking more time for them to develop new friendship, because of the repeating in Secondary 2 or adapting to a new school. More attention and further study are warranted in relation to the behavioral intention of alcohol consumption in this group.

Subjective outcome evaluation highlighted positive results from all participants. Although the longitudinal data from the objective outcome evaluation did not support any improvement, the effect of the program was found to be positive to the new comers in the junior secondary years. Therefore, in conjunction with the previous findings and based on the objective outcome evaluation, the Secondary 2 program is effective, particularly for new comers of the program. There are several limitations of this study. Firstly, only two schools are involved and the sample size was relatively small, raising the potential for sample bias, and it makes any generalization of the findings difficult. Secondly, the present study is based on a one-group pre- /posttest design which may not be the most appropriate. Other approaches such as the randomized control trial which can provide a more rigorous design to give more insight on the effectiveness of intervention program could have been considered. Thirdly, a comparative portion of students dropped out of the program which may affect longitudinal observation and observation of long-term effects. Finally, if the program is more beneficial to those with below average academic performances, and if some of them may have to repeat studying in Secondary 1 and lose the chance to be followed up, the evaluation of the program will be incomplete. Nevertheless, with the current paucity of youth studies in Macau, the present study can contribute to the understanding of the potential benefits of evidence-based youth work and provide further baseline data for evaluation of Secondary 3 periods in the future.

## Figures and Tables

**Table 1:** Number of participants and completed questionnaires collected at year 1 (Wave 1 and Wave 2) and year 2 (Wave 3 and Wave 4).

	Year 1		Year 2	
	Wave 1	Wave 2	Wave 3	Wave 4
Cases of 2 schools	239	242	268	244
Successfully match		232		236
Old participants			189	173
New participants			79	63

**Table 2:** Participant characteristics of P.A.T.H.S. Secondary 2 old and new students. SSF: Social security fund.

Variables	Old (*n* = 189) *n* (%)	New (*n* = 79) *n* (%)	Total (*n* = 268) *n* (%)	*P* value
Gender				
Male	120 (63.8)	45 (57.0)	165 (61.8)	0.292
Female	68 (36.2)	34 (43.0)	102 (38.2)	
Age				
12	5 (2.6)	0 (0.0)	5 (1.9)	
13	86 (45.5)	0 (0.0)	86 (32.1)	
14	50 (26.5)	21 (26.6)	71 (26.5)	**0.000**
15	28 (14.8)	37 (46.8)	65 (24.3)	
≥16	20 (10.6)	21 (26.6)	41 (15.3)	
Family members				
1	1 (0.5)	0 (0.0)	1 (0.4)	
2	6 (3.2)	7 (9.2)	13 (4.9)	
3	40 (21.2)	18 (23.7)	58 (21.9)	0.349
4	90 (47.6)	35 (46.1)	125 (47.2)	
5	34 (18.0)	12 (15.8)	46 (17.4)	
≥6	18 (9.6)	4 (5.2)	22 (8.3)	
Parental marriage status				
Divorced	20 (10.7)	8 (10.5)	28 (10.6)	
Separated	6 (3.2)	4 (5.3)	10 (3.8)	0.712
Married	152 (81.3)	58 (76.3)	210 (79.8)	
Family happiness				
Very unpleasant	12 (6.4)	8 (10.4)	20 (7.5)	
Unpleasant	19 (10.1)	15 (19.5)	34 (12.8)	
General	74 (39.4)	26 (33.8)	100 (37.7)	0.164
Pleasant	57 (30.3)	21 (27.3)	78 (29.4)	
Very pleasant	26 (13.8)	7 (9.1)	33 (12.5)	
SSF				
Yes	15 (8.0)	5 (6.5)	20 (7.6)	0.689
No	172 (92.0)	71 (92.2)	243 (92.0)	

**Table 3:** Findings from the subjective outcome evaluation (*n* = 257).

	Your views towards the course(s)	Percentage of responses (%)							
		1	2	3	A	4	5	6	B
1	The objectives of the curriculum are very clear.	0.4	6.5	13.8	20.7	31.0	39.1	7.3	77.4
2	The design of the curriculum is very good.	1.9	6.1	15.3	23.4	37.9	33.0	4.2	75.1
3	The activities were carefully planned.	1.5	6.9	17.6	26.1	33.7	33.3	5.0	72.0
4	The classroom atmosphere was very pleasant.	4.2	6.1	14.9	25.3	34.1	28.0	10.3	72.4
5	There was much peer interaction amongst the students.	3.8	7.7	11.9	23.4	28.7	31.0	13.0	72.8
6	I participated actively during lessons (including discussions, sharing, games, etc.).	1.9	6.1	11.9	19.9	38.7	28.0	11.1	77.8
7	I was encouraged to do my best.	3.8	8.0	15.3	27.2	37.2	27.6	5.4	70.1
8	The learning experience I encountered enhanced my interest towards the lessons.	3.4	6.5	15.7	25.7	36.0	29.5	6.5	72.0
9	Overall speaking, I have very positive evaluation of the program.	3.8	10.0	14.2	28.0	41.4	23.0	4.6	69.0
10	On the whole, I like this curriculum very much.	7.7	6.5	16.1	30.3	30.7	28.0	8.4	67.0

	Your views towards the instructor(s)								
1	The instructor(s) had a good mastery of the curriculum.	0.8	4.6	13.0	18.4	27.6	39.5	13.0	80.1
2	The instructor(s) was well prepared for the lessons.	0.8	3.4	11.1	15.3	29.5	36.4	17.2	83.1
3	The instructor(s)' teaching skills were good.	1.9	5.0	11.9	18.8	28.7	39.5	11.5	79.7
4	The instructor(s) showed good professional attitudes.	1.9	2.7	8.8	13.4	31.0	36.4	17.2	84.7
5	The instructor(s) was very involved.	0.8	3.4	11.5	15.7	26.4	37.9	18.0	82.4
6	The instructor(s) encouraged students to participate in the activities.	0.4	3.1	8.0	11.5	29.9	35.2	21.5	86.6
7	The instructor(s) cared for the students.	0.0	3.8	10.0	13.8	25.3	37.2	22.2	84.7
8	The instructor(s) was ready to offer help to students when needed.	0.4	3.8	7.3	11.5	27.2	39.5	19.2	85.8
9	The instructor(s) had much interaction with the students.	1.1	5.7	11.5	18.4	26.1	35.6	18.4	80.1
10	Overall speaking, I have very positive evaluation of the instructors.	1.9	5.7	7.7	15.3	26.8	35.6	20.7	83.1

Remarks: 1 = strongly disagree, 2 = disagree, 3 = slightly disagree, 4 = slightly agree, 5 = agree, 6 = strongly agree.

A = sum of the disagree responses (1 + 2 + 3), B = sum of the agree responses (4 + 5 + 6).

**Table 4:** Perceptions on the extent to which the course has helped them (*n* = 257).

	The extent to which the course has helped you	Percentage of responses (%)						
		1	2	A	3	4	5	B
1	It has strengthened my bonding with teachers, classmates, and my family.	8.8	20.3	29.1	41.0	25.7	2.3	69.0
2	It has strengthened my resilience in adverse conditions.	8.0	18.4	26.4	37.9	25.7	8.0	71.6
3	It has enhanced my social competence.	5.7	20.7	26.4	33.7	31.0	6.9	71.6
4	It has improved my ability in handling and expressing my emotions.	7.7	15.3	23.0	39.8	26.4	8.0	74.3
5	It has enhanced my cognitive competence.	8.4	13.4	21.8	36.8	30.7	9.2	76.6
6	My ability to resist harmful influences has been improved.	8.8	16.5	25.3	36.4	26.8	9.6	72.8
7	It has strengthened my ability to distinguish between the good and the bad.	7.3	14.6	21.8	35.6	31.4	9.2	76.2
8	It has increased my competence in making sensible and wise choices.	7.3	13.8	21.1	37.9	29.9	9.6	77.4
9	It has helped me to have life reflections.	10.0	13.0	23.0	32.2	29.9	12.6	74.7
10	It has reinforced my self-confidence.	13.4	13.0	26.4	35.2	25.7	10.7	71.6
11	It has increased my self-awareness.	10.0	16.1	26.1	32.6	29.1	10.0	71.6
12	It has helped me to face the future with a positive attitude.	7.7	15.3	23.0	37.2	28.7	8.0	73.9
13	It has helped me to cultivate compassion and care about others.	8.0	14.2	22.2	37.5	30.7	6.5	74.7
14	It has encouraged me to care about the community.	9.2	19.5	28.7	36.0	25.7	7.3	69.0
15	It has promoted my sense of responsibility in serving the society.	8.0	20.3	28.4	33.3	29.5	6.9	69.7
16	It has enriched my overall development.	10.3	13.8	24.1	37.5	26.8	9.6	73.9

Remarks: 1 = unhelpful, 2 = not very helpful, 3 = slightly helpful, 4 = helpful, 5 = very helpful.

A = sum of the unhelpful responses (1 + 2), B = sum of the helpful responses (3 + 4 + 5).

**Table:** (a)

		1	2	A	3	4	B
3	If your friends have needs and conditions similar to yours, will you suggest him/her to join this course?	11.9	21.8	33.7	51.7	11.5	63.2
4	Will you participate in similar courses again in the future?	16.1	29.1	45.2	42.9	8.4	51.3

Remarks: 1 = definitely will not, 2 = will not, 3 = will, 4 = definitely will.

A = sum of those will not (1 + 2), B = sum of those will (3 + 4).

**Table:** (b)

		1	2	3	A	4	5	6	B
5	On the whole, are you satisfied with this course?	4.6	3.8	14.9	23.3	46.0	19.9	8.4	74.3

Remarks: 1 = very dissatisfied, 2 = moderated dissatisfied, 3 = slightly dissatisfied, 4 = satisfied, 5 = moderately satisfied, 6 = very satisfied.

A = sum of those will not satisfy (1 + 2 + 3), B = sum of those will satisfy (4 + 5 + 6).

**Table 6:** The changes in the Secondary 2 program participants based on the different scale.

	Pretest		Posttest		*t*-value	*P* value
	Mean	SD	Mean	SD		
CYPDS	4.33	0.58	4.28	0.61	1.88	0.062
LIFE	3.66	1.01	3.70	1.13	−0.57	0.568
SA	3.01	0.67	2.96	0.72	1.038	0.300
BI	1.54	0.55	1.64	0.54	−3.13	**0.002**

Note: Significant *P* values are in bold.

**Table 7:** Comparison of the old and new participants in Wave 3 data.

	Old (*n* = 189)		New (*n* = 79)		*t*-value	*P* value
	Mean	SD	Mean	SD		
BO subscale	4.59	0.74	4.38	0.86	2.024	**0.044**
RP subscale	4.14	0.92	3.86	0.93	2.268	**0.024**
EC subscale	4.21	0.85	3.92	0.88	2.526	**0.012**
CC subscale	4.35	0.81	4.12	0.78	2.148	**0.033**
ID subscale	4.07	0.79	3.74	0.77	3.191	**0.002**
BF subscale	4.21	0.89	3.96	0.79	2.137	**0.033**
PI subscale	4.21	0.93	3.87	1.03	2.671	**0.008**
SP subscale	4.76	1.25	4.31	1.30	2.671	**0.008**
CYPDS	4.34	0.59	4.14	0.61	2.513	**0.013**
LIFE	3.77	1.04	3.28	0.83	3.735	**0.000**
SA	3.07	0.61	2.85	0.84	2.071	**0.041**
BI	1.52	0.56	1.65	0.53	1.813	0.071
BI (item 1)	2.13	1.10	2.54	1.07	2.807	**0.005**
BI (item 2)	1.35	0.70	1.44	0.83	0.814	0.417
BI (item 4)	1.39	0.70	1.51	0.79	1.164	0.247

Note: BO: bonding, RP: recognition for positive behavior subscale, EC: emotional competence subscale, CC: cognitive competence subscale. ID: clear and positive identity subscale, BF: beliefs in the future subscale, PI: prosocial involvement subscale, SP: spirituality subscale, CPYDS: mean of the 15 subscales, LIFE = life satisfaction scale, SA: school adjustment measures, BI: behavioral intention scale, BI (item 1): will you drink alcohol in the coming 2 years? BI (item 2): will you smoke cigarettes in the coming 2 years? BI (item 4): will you have sex in the future 2 years? Significant *P* values are in bold.

**Table 8:** The changes of the new participants based on the different objective indicators.

	Pretest		Posttest		*t*-value	*P* value
	Mean	SD	Mean	SD		
New participants (*n* = 63)						
BO subscale	4.55	0.71	4.31	0.80	2.380	**0.020**
RP subscale	3.96	0.90	3.94	0.89	0.126	0.900
EC subscale	3.98	0.84	4.24	0.98	2.402	**0.019**
CC subscale	4.22	0.72	4.44	0.80	2.418	**0.019**
ID subscale	3.79	0.78	4.08	0.80	3.023	**0.004**
BF subscale	4.06	0.83	3.86	0.74	1.759	0.083
PI subscale	4.00	0.99	4.05	0.95	0.344	0.732
SP subscale	4.45	1.27	4.63	1.21	1.218	0.228
CYPDS	4.24	0.57	4.27	0.55	0.532	0.596
LIFE	3.32	0.81	3.62	1.09	2.138	**0.037**
SA	2.81	0.77	3.07	0.84	2.324	**0.023**
BI	1.62	0.50	1.91	0.51	4.376	**0.000**
BI (item 1)	2.57	1.06	2.90	1.00	2.490	**0.005**
BI (item 2)	1.35	0.74	1.70	0.91	3.017	**0.004**
BI (item 4)	1.49	0.78	1.94	1.00	3.727	**0.000**

Note: BO: bonding, RP: recognition for positive behavior subscale, EC: emotional competence subscale, CC: cognitive competence subscale. ID: clear and positive identity subscale, BF: beliefs in the future subscale, PI: prosocial involvement subscale, SP: spirituality subscale, CPYDS: mean of the 15 subscales, LIFE: life satisfaction scale, SA: school adjustment measures, BI: behavioral intention scale, BI (item 1): will you drink alcohol in the coming 2 years? BI (item 2): will you smoke cigarettes in the coming 2 years? BI (item 4): will you have sex in the future 2 years? Significant *P* values are in bold.
